# Field-Programmable Gate Array-Based Implementation of Zero-Trust Stream Data Encryption for Enabling 6G-Narrowband Internet of Things Massive Device Access

**DOI:** 10.3390/s24030853

**Published:** 2024-01-28

**Authors:** Wen-Chung Tsai

**Affiliations:** Department of Intelligent Production Engineering, National Taichung University of Science and Technology, Taichung City 404, Taiwan; azongtsai@nutc.edu.tw

**Keywords:** hardware acceleration, information security, Internet of Things, one-time password, one-time pad, zero-trust network

## Abstract

With the advent of 6G Narrowband IoT (NB-IoT) technology, IoT security faces inevitable challenges due to the application requirements of Massive Machine-Type Communications (mMTCs). In response, a 6G base station (gNB) and User Equipment (UE) necessitate increased capacities to handle a larger number of connections while maintaining reasonable performance during operations. To address this developmental trend and overcome associated technological hurdles, this paper proposes a hardware-accelerated and software co-designed mechanism to support streaming data transmissions and secure zero-trust inter-endpoint communications. The proposed implementations aim to offload processing efforts from micro-processors and enhance global system operation performance by hardware and software co-design in endpoint communications. Experimental results demonstrate that the proposed secure mechanism based on the use of non-repeating keys and implemented in FPGA, can save 85.61%, 99.71%, and 95.68% of the micro-processor’s processing time in key block generations, non-repeating checks, and data block transfers, respectively.

## 1. Introduction

With the development of 6G technology applications, the future Internet of Things (IoT) will face an increasing number of endpoint devices that need to simultaneously transmit data to each other. For example, applications integrating edge and fog computing with the Internet of Intelligence may require the use of Virtual Network Functions (VNFs) [1], in which there are many concerns about information security during the transmission process [2,3]. At this point, a significant challenge for research and industry is whether IoT endpoint devices can provide sufficient transmission efficiency and adequate security measures to handle the simultaneous data transmission required by various IoT applications. Given the diverse nature of IoT applications and the need for simultaneous transmission among a large number of endpoints, this paper is based on the Double OTP (D-OTP) proposed in [4], and then furthermore provides a zero-trust network architecture, namely the One-Time Password Pad Process (OTP^3^). This aims to realize a secure mechanism for transmitting asynchronous streaming signals between IoT device endpoints. Compared to pure software design, utilizing hardware acceleration can significantly enhance system performance.

This research was inspired by these reports. Ref. [5] mentioned that a smartwatch designed for children has been found to have cybersecurity vulnerabilities, enabling hackers to steal the children’s location. Moreover, the Mirai zombie virus [6], known for large-scale attacks on IoT devices, primarily targeted home routers, network cameras, and smart TVs at the time. Subsequently, eleven new variants of the virus were discovered, notably one that targeted wireless projection servers used in offices. This event serves as a reminder that hackers can infiltrate corporate networks and steal business secrets through insecure commercial devices. Furthermore, in an analysis reported by the National Aeronautics and Space Administration (NASA) [7], their Jet Propulsion Laboratory (JPL) was breached by hackers using a Raspberry Pi device as a pivot point. The hackers successfully invaded the laboratory, stealing critical data, making it one of the most prominent cybersecurity events worldwide in recent years. It was known that Industrial Information System (IIS) or Industrial Control System (ICS) of the enterprise organization had been attacked and might be extorted by hackers for a high ransom [8,9], and thus security will become the most mainstream development initiative.

In the post-5G era, the concept of information security protection through methods including zero-trust are rapidly gaining prominence [10]. The zero-trust network requires IoT devices to ensure their own security. However, due to the limited computing capability and hardware resources of IoT devices, conventional IoT devices often fail to provide adequate self-security. This vulnerability becomes more pronounced when considering the future threat of quantum computing. The advancement in the Internet of Things will be accelerated by the 5G system [11] and the coming 6G technology, which includes considerations about whether enough security measures can be provided when utilizing NB-IoT (Narrow Band-Internet of Things) technology [12] to meet the application requirements of Massive Machine-Type Communications (mMTC) [13]. It is evident that the opportunities in the IoT landscape are rapidly growing. However, this growth also raises security concerns for devices and applications. At this point, a significant challenge for research, education, and industry is whether IoT endpoint devices can simultaneously provide adequate transmission efficiency and comprehensive security measures [14].

Facing this issue and challenge, this paper is based on the functionality and design required for the proposed OTP^3^ operation process, which aims to realize a secure mechanism suitable for transmitting asynchronous streaming signals between IoT device endpoints. This mechanism adopts a fog computing architecture [1]. The cloud server is responsible for control plane operations and necessary management settings related to transmission security, such as key exchange, with various endpoint devices (i.e., fog nodes). Each endpoint device conducts routine processing tasks like data compression/decompression, encryption/decryption, and transmission/reception in the data-plane. This approach significantly reduces the storage and computational burden on the server. In terms of system implementation, the design and management functionalities need to be updated and flexible in the control plane, so development will primarily utilize a software-centric approach. Additionally, in the data-plane, considering the routine, repetitive, and cyclic nature of data-flow processing, Field-Programmable Gate Array (FPGA) will be employed for necessary hardware data stream processing pipelines to ease the computational load on the device’s micro-processor (µP).

In addressing the security requirements of zero-trust IoT devices in future 6G communication, the primary contribution of this paper is the attainment of theoretical un-breakability based on one-time pad [15] through the proposed secure mechanism. A one-time pad is one in which a key can only be used once (i.e., non-repeating) for data encryption, which is proven by Shannon [16] to possess theoretical un-breakability. Nevertheless, the computational intensity of the non-repeating check poses a challenge for conventional pure software implementations. Consequently, the proposed secure mechanism incorporates purpose-designed hardware accelerators to offload the computational intensity from the software executed by the micro-processor. As a result, the integration approach of the micro-processor and hardware accelerators in the FPGA, encompassing system design architecture, execution flow, and bus components of the micro-processor, accelerators, and other essential controllers, will be elucidated in the Section 4 “Design Implementation”.

This paper is structured as follows: Section 2 provides an explanation of the background of zero-trust networks, IoT security architecture, and the proposed mechanism. Section 3 introduces the research methodology, encompassing the applied cipher algorithm, security method, and operational process. Section 4 delves into the system design and implementation. In Section 5, experimental results are analyzed. Finally, Section 6 presents a discussion and the conclusion.

## 2. Background

This section begins by outlining the motivation behind enhancing IoT security for stream data transmission. We then delve into an exploration of the background of zero-trust network architecture, with related security issues and challenges. Additionally, we highlight the constraints associated with conventional IoT designs and implementations, with a specific focus on comparing various existing hardware-accelerated processing techniques. Finally, we provide an overview of the functions in prior work which serves as the foundation for this research.

### 2.1. Motivation to Improve IoT Security for Stream Data Transmission

Common IoT devices include various sensors such as sound, image, gas, pressure, temperature, and motion sensors, providing applications for environmental monitoring, disaster prevention, rescue operations, and so on. Considering the sensors mentioned above, if used in applications like calculating water, electricity, and gas flow, fleet route monitoring, data analyses in production lines, illegal logging detection in forests, and collection of biomedical signals from patients, which involve sensitive, confidential, and private data, the need for a secure transmission mechanism providing information security is crucial in such applications. We observed a common characteristic among these sensors, which is that they mostly provide continuous output in the form of streaming signals. Therefore, integrating real-time encryption and decryption into the processing of streaming signals is expected to enhance their effectiveness and efficiency. It is evident that the opportunities for IoT security are rapidly growing. Consequently, the following will sequentially explain the research background and current developments related to IoT security, as well as the approach taken in this paper.

### 2.2. Zero-Trust Network Architecture and Future IoT Applications

**Figure 1:**
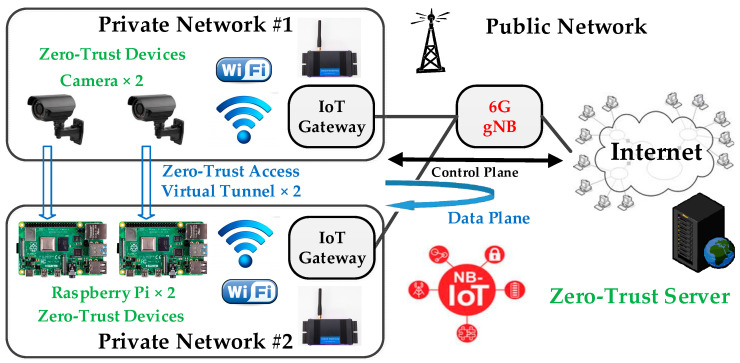
Potential applications of zero-trust network devices with 6G NB-IoT endpoint devices.

As shown in Figure 1, a zero-trust system, such as the NB-IoT architecture, typically consists of endpoint devices (such as various sensor nodes), network equipment (such as IoT gateways and 6G base stations), core networks, and cloud application servers. Users can access endpoint devices directly via the network or indirectly access data sent to the cloud server through the endpoint devices. Accordingly, these endpoint devices are not always subject to the control of a firewall and VPN server, making the concept of zero-trust network an effective solution to address this security concern. In line with this, the US National Institute of Standards and Technology (NIST) introduced the SP800-207 zero-trust architecture guidelines in 2019 [17], urging enterprises to adopt zero-trust information security strategies to combat threats like ransomware attacks. Furthermore, the US federal government mandated that all government agencies must transition to a zero-trust architecture for their security strategies by the end of 2024 [18].

In reference to “Figure 3: Zero Trust Access process” from the *Network and Security Strategy* published by the Government of Canada [19], devices initially need to submit access requests to an authentication service. Upon validation of the request, the devices can proceed with the requests, initiating the data-flow session through an encrypted micro-segmented tunnel that remains active only for the duration of the session. Additionally, based on the concept of zero-touch in the zero-trust network [10], security in the network devices such as gateways, base stations, and core networks will no longer be trusted. In this scenario, the security mechanism for network access must be provided by the IoT devices themselves.

As depicted in Figure 1, a pair of communicating zero-trust devices consists of a camera and a Raspberry Pi, situated in separate private networks. In order to initiate a data-flow session, the communicating devices must send a request to the “Zero-Trust Server” through the control plane over the public network. Upon approval of the request, a secure “Zero-Trust Access Virtual Tunnel” is established. Subsequently, the 6G gNB facilitates the forwarding of video stream data via the data plane. This mechanism allows the creation of two or more “Zero-Trust Access Virtual Tunnels” for multiple paired zero-trust devices between two distinct private networks or within the same private network. Accordingly, the key blocks used for communication must be synchronously updated by the communicating paired devices. In this way, during the data transmission process, there is no need for additional protection through the network devices, effectively realizing the zero-touch concept required by the zero-trust network.

**Figure 2:**
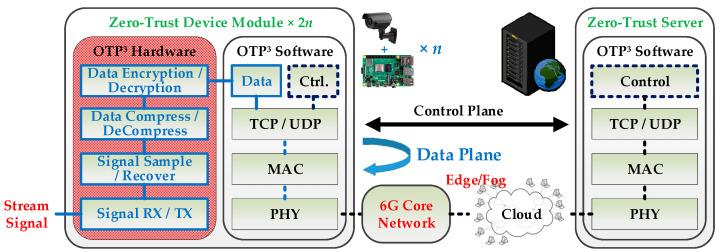
OTP^3^ IoT cybersecurity system facilitating data exchange at the network edge.

As Figure 2 shows, concerning data processing in the IoT, in contrast to cloud computing where data is sent from scattered devices to centralized ones (such as cloud servers), the fog computing architecture handles data in a relatively decentralized manner, with data computation and storage located closer to the network edge. In this architecture of edge computing collaborating with cloud and fog, since the data transmission process can bypass or avoid the existing core network system, it can reduce core network resource usage, lower transmission latency, enhance transmission speed, and improve user experience. However, because the system management of the existing core network may be bypassed during data exchange and due to the interconnection of a large number of low-cost IoT devices, this IoT transmission architecture based on cloud and fog computing raises concerns about information security. For more information regarding the implementations of security mechanisms and data-flow processes, the following sub-sections in Section 3 “Research Methodology” will provide the details.

### 2.3. Limitations in Conventional IoT Protocols and Architectures

**Figure 3:**
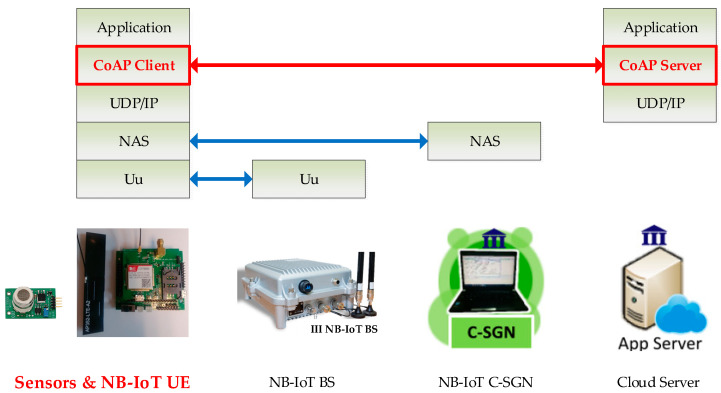
NB-IoT sensor system employs CoAP as the IoT communication protocol.

Regarding the limitations of traditional IoT architectures, based on previous research findings in [4], these include the ability to transmit data collected by sensors to the Internet through an NB-IoT User Equipment (UE) of sensor hub, as shown in Figure 3. The data is sent using the Constrained Application Protocol (CoAP) transport protocol, and messages are exchanged through the Internet with the application cloud server [20]. CoAP, defined by the Internet Engineering Task Force (IETF) [21], is a lightweight transport protocol used for data exchange at the application layer of devices with constrained resources, such as IoT terminal devices. However, the CoAP protocol includes the ability to use Datagram Transport Layer Security (DTLS) for encrypted data transmission. However, this NB-IoT sensor hub utilizes a low-cost 8-bit micro-processor [22], with an operating frequency of only 16 MHz and a program storage capacity of only 64 Kbytes. In such resource-constrained device applications, achieving assured transmission security is indeed a challenge beyond mere transmission functionality.

Accordingly, the highly demanding yet more challenging issues for developing the NB-IoT communication system are as follows:

1. The first issue to address is the efficiency of the micro-processor’s execution. Scholars in study [23] concluded the following: “Applying our model, we identified the cryptographic operations being the main cost factor”. In other words, cryptographic operations, in the absence of hardware accelerator support, are the main performance cost that must be borne. This might be difficult for low-cost IoT devices to handle additional computational burdens and power consumption requirements, especially for achieving the desired continuous streaming data transmission functionality in this paper.
2. The second issue is the operation mode of CoAP, which is a client–server architecture. In other words, to achieve network transmission functionality between device endpoints, all data transmission processes must go through CoAP proxy servers. Moreover, all application layer data forwarded by the proxy must be received and decrypted by the server before being encrypted and sent out again. During these processes, numerous information security concerns arise. For example, plaintext data used when establishing connections between devices and servers may be intercepted. Furthermore, proxy servers are easily targeted for hacker attacks [24].

To provide security functionalities for IoT devices, manufacturers are increasingly aware of the need to begin investing in this area. However, due to the diverse types of devices and varying security setup options, the lack of global standards for device and application-layer security poses significant challenges in securing the application layer of devices. This has left device manufacturers and IoT deployment companies confused and hesitant. To solve this problem, a leading cybersecurity company, Trend Micro, has introduced Trend Micro IoT Security (TMIS) 3.0, an IoT device protection software [25]. TMIS is a security solution that can run on Raspberry Pi [26], designed for embedded IoT devices communicating externally using Internet Protocol (IP). TMIS monitors and protects these devices to guard against various potential risks, including data theft and ransomware attacks. Given its system’s hardware resource requirements, TMIS is clearly not suitable for running on our used NB-IoT UE Sensor Hub, as shown in Figure 3. Furthermore, upon further investigation, we understand that the TMIS solution provided by Trend Micro does not offer complementary hardware acceleration support. Therefore, careful consideration is needed regarding the computational resources of the micro-processor that the software requires to run on IoT devices, especially for routine data encryption and decryption operations that this paper addresses. For more detailed information regarding the use of CoAP protocol in the IoT sensor system, please refer to the previous work [4] with respect to this paper.

### 2.4. Development of IoT Security Mechanisms with Hardware Accelerators

Regarding IoT security approaches with hardware acceleration support, ARM has initiated a project named Beetle [27]. We noticed that the True Random Number Generator (TRNG) module provided by the platform is connected to the Cortex-M3 processor through the AXI/AHB bus. Therefore, when executing the Transport Layer Security (TLS) protocol [28], the micro-processor can indirectly choose to utilize the assistance of the TRNG hardware module to enhance network security performance. Furthermore, ARM has introduced the Platform Security Architecture (PSA) [29] to bring a unified approach to IoT security in the industry. In comparison to ARM’s approach of providing hardware acceleration modules for buses to the micro-processor, this paper proposes an innovative method based on achieving secure transmission of “streaming signals” as the application goal. According to the limitations of the above-mentioned methods, we directly place the hardware security module within the “data stream processing pipeline.” This further increases the data processing throughput per unit time and reduces the software computational burden on the micro-processor. In addition to the solution provided by ARM, the current state of achieving IoT security 0n the market is in a state of diverse opinions and approaches. This also highlights a potential severe talent gap crisis in future IoT security needs. Facing this issue and challenge, this paper outlines the architectural approach for a security mechanism suitable for transmitting “asynchronous streaming signals between IoT device endpoints.”

Additionally, the research challenges of applying FPGA to accelerate the processing of endpoint communications and secure mechanisms are summarized as follows: Mujahid and Ullah introduced a partial pattern classification system employing Content-Addressable Memory (CAM) on FPGA [30]. Similarly, in this paper, CAM is utilized to accelerate the comparison process of key blocks. Chaudhry et al. present an approach that leverages serverless computing to integrate Multiaccess Edge Computing (MEC) and Virtual Network Function (VNF) at the system level [31]. They further investigate improving resource utilization by leveraging FPGA-enabled MEC servers for a high-definition real-time video streaming application. In contrast to the FPGA-enabled MEC servers, the proposed OTP^3^ mechanism is implemented in an IoT device. Goswami et al. present a SNOW 3G crypto processor architecture, a stream cipher algorithm used for 4G LTE, designed for security applications with a focus on area, power, and efficiency [32]. The distinction with regard to the proposed mechanism lies in OTP^3^’s goal of supporting secure streaming data transmissions for zero-trust inter-endpoint communications in future 6G networks. Szymanski presents deterministic packet switches (D-switches) used in the control-plane of Software-Defined Networking (SDN) [33]. The difference with the proposed mechanism is that OTP^3^ deals with stream data encryption in the data-plane. Both mechanisms share common objectives, emphasizing the potential for cost reduction, energy efficiency, and high capacity, as seen in previous research [34,35].

### 2.5. One-Time Password, One-Time Pad, and D-OTP

A one-time password is a password that is valid for only one login session on a computer or for a single data transfer over a network [36]. Through implementation of a one-time password ecosystem, the static symmetric-key authentication-related shortcomings of the public key can be avoided. In real applications, OTP can be repeating, due to the limited length of numbers. Furthermore, a one-time pad is one in which a key can only be used once (i.e., non-repeating) for encrypted operations [15]. One-time pad is an encryption method in classical cryptography and has been reported by Shannon [16] to be theoretically unbreakable. In the previous research [4], we proposed and implemented our own proposed key update mechanism, which can automatically execute both one-time password (for changing password) and one-time pad (for non-repeating key) in IoT devices as a network communication pair, namely D-OTP. However, a limitation was the poor communication latency overhead, incurred through application of the D-OTP mechanism. Therefore, a hardware accelerated processing is required, as introduced in this paper. As they are introduced, these challenges will be explicitly addressed within our proposed design, and a comprehensive insight into this design methodology is presented in the forthcoming section.

### 2.6. Methods for Implementing Streaming Data Encryption

In the selection of data encryption methods, stream cipher is a type of symmetric-key encryption algorithm, meaning both encryption and decryption parties use the same key. During encryption, a key stream is generated using a pseudorandom number generator, and this key stream is sequentially encrypted with a plaintext data stream to obtain a ciphertext data stream. In terms of design, the stream cipher method is relatively straightforward, allowing real-time transmission effects during network transmission. It is generally limited in its application to streaming data transmission, such as audio and video, and typical stream cipher algorithms include RC4 and eStream [37,38]. On the other hand, block cipher is also a type of symmetric-key encryption algorithm. The difference is that block encryption requires the plaintext data to be divided into several equally sized blocks before performing matrix operations on the master key and the first plaintext block to obtain a ciphertext block. It also simultaneously generates the round key needed for the calculation of the next set of plaintext blocks. Due to its relatively complex operational design and higher computing performance requirement, block cipher can be used for streaming data transmissions, but is more suitable for transferring file data. Typical block cipher algorithms include DES and AES [39,40]. After comparing the advantages and disadvantages of stream cipher and block cipher, this study chooses stream cipher as the encryption method to implement the proposed mechanism in this paper.

### 2.7. Discussion on the Security of Stream Cipher Algorithms

In terms of network transmission security, as far as we understand, vulnerabilities that may be exploited in the system primarily exist in the standards and protocols of the applied encryption algorithms. Taking RC4 as an example, it is a stream cipher algorithm that has become part of some commonly used standards and protocols. For instance, it has been included in the IEEE 802.11 standard [41], pertaining to the network link layer protocols Wired Equivalent Privacy (WEP) and Wi-Fi Protected Access (WPA) [42]. It has also been incorporated into the IETF-RFC standard, relating to the network transport layer protocols Secure Sockets Layer (SSL) and Transport Layer Security (TLS). Due to concerns about potential vulnerabilities [43], IETF-RFC-7465 announced the discontinuation of the use of the RC4 encryption algorithm in TLS [44]. However, based on our understanding, the vulnerabilities that may be exploited in the system primarily lie within the operations of encryption protocol (i.e., WEP), rather than the applied encryption algorithm itself (i.e., RC4). Therefore, this paper focuses on using the RC4 algorithm just as a Pseudo Random Number Generator (PRNG), coupled with the OTP^3^ non-repeating key mechanism to realize an efficient streaming encryption mechanism suitable for the IoT environment. The relevant results will be explained in the Section 5 “Experimental Results” and Section 6 “Discussion and Conclusions”.

## 3. Research Methodology

This section introduces the proposed OTP^3^ operational process as a mechanism that aligns with the principles of zero-trust network access.

### 3.1. Methods Proposed for Enhancing IoT Data Transmission Security

Stream encryption is a symmetric-key encryption algorithm, where both encryption and decryption parties use the same key. The sender utilizes this key as a seed for a Pseudo Random Number Generator (PRNG) [45] to generate a key stream. This key stream is then sequentially used to encrypt a plaintext data stream, resulting in a ciphertext data stream. The receiver can decrypt the data if they possess the same key. Building upon traditional encryption methods, our research introduces a method for secure IoT communication, referred to as D-OTP, based on the previous work [4].

D-OTP combines two aspects: “Operation Password” and “Encryption Key”, briefly explained as follows: (1) The “Operation Password” refers to a method for users to utilize to gain system access. One-Time Password (hereafter referred to as OTP password) is a single-use password used by the user during login. OTPs are widely used security authentication tools, commonly seen in financial, telecom, and online gaming transactions, and the applications applied in [46]. (2) The “Encryption Key” includes critical data used for computational operations with plaintext data to obtain ciphertext data within the system. One-Time Pad (hereafter referred to as OTP-key) is a key that can only be used once during encryption operations, and OTP-key is a classical encryption algorithm in cryptography and is proven by Shannon [16] to possess theoretical un-breakability. For zero-trust network device access, this paper’s innovation lies in further integrating OTP password and OTP-key with the “process of hardware accelerated stream data encryption”, namely the One-Time Password Pad Process (OTP^3^), which aims to fulfill the future stringent confidentiality requirements for zero-trust secure data transmission [47]. The detailed specification of OTP^3^ will be further explained in the following section.

### 3.2. Security Guarantee Scheme and Operation of OTP^3^

**Figure 4:**
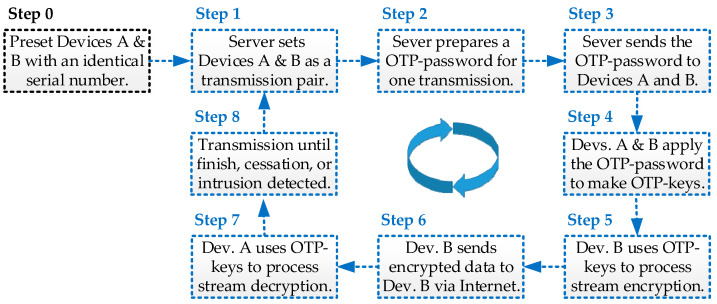
An OTP^3^ operation flow for transmitting stream data between two paired devices.

The proposed zero-trust stream data encryption mechanism is based on the advantages of integrating “One-Time Password (OTP password)” and “One-Time Pad (OTP-key)” for “processing secure stream data transmission”, named OTP^3^. This will meet the following three requirements for meeting the un-breakable security guarantee by Shannon [16]: (1) the key must be generated randomly, (2) promise that the key is not reused, and (3) ensure secure distribution of the key to both encryption and decryption endpoint devices. Accordingly, the operation flow of OTP^3^ is introduced as follows:

As Figure 4 shows, the OTP^3^ operation process includes several steps, and each step is briefly described in sequence, as follows: Step 0: The User presets an identical Serial Number (SN) to both Device A and Device B as a communication pair, and the used SN is essentially non-repeating in other communication pairs, as defined in the previous work of D-OTP [4]. Step 1: The Server intends to have Device A receive the streaming signal detected by its paired Device B. Step 2: The server generates a non-repeating OTP password, as introduced in [4]. Step 3: The server uses public network encryption technologies (such as SSL/TLS) and symmetric-key exchange techniques like the classic Diffie–Hellman (DH) [48] to transmit this OTP password to the paired devices A and B. Step 4: Devices A and B each generate non-repeating OTP keys capable of meeting cryptographic security standards using this OTP password and the preset SN. Step 5: Device B continues to encrypt the streaming signal it inputs using these OTP keys. Step 6: Device B continues to transmit the encrypted ciphertext generated by the encryption process to Device A via the public network (no need for further encryption during the process). Step 7: Device A continues to decrypt the network data it inputs using these OTP keys. Step 8: Until the transmission is completed, stopped, or an external intrusion signal is detected, the OTP password and OTP keys used for this transmission are discarded, and the process returns to Step 1 to prepare for the start of another transmission.

It is worth noting that, in Step 6, during the transmissions between Device A and Device B, there is no additional encryption operation required by any network equipment (such as gateway, router, and core network). Accordingly, the proposed OTP^3^ mechanism is aligning with the principle of zero-trust network access.

### 3.3. Security Implications of OTP^3^

The security implications can be analyzed in the setup plane, control plane, and data plane, as introduced below:

#### 3.3.1. Setup Plane

Referring to Figure 1, the zero-trust network system essentially comprises a “Zero-Trust Server” and multiple zero-trust devices, each equipped with at least one “Zero-Trust Device Module”, as depicted in Figure 2. During device deployment, users can preconfigure two modules with identical Serial Numbers (SNs) to form a communication pair, ensuring that the used SN does not repeat in other communication pairs (refer to Step 0 in Figure 4). In operations, the SN is employed to generate a non-repeating key for each transmission (refer to Step 3 in Figure 4). Unlike traditional Pre-Shared Key (PSK) methods, the SN serves as an initial PSK but is not transmitted over the public network. In practice, the SN is stored in a Read-Only Memory (ROM), making it accessible only by directly reading the ROM in the device module. Moreover, even if the SN of a specific zero-trust module becomes known to a third party, the one-time use principle prevents its utilization in compromising other device modules.

#### 3.3.2. Control Plane

The control plane is dedicated to system management during the operational stage. Prior to initiating data transmission between two paired zero-trust modules, the modules are required to seek permission from the “Zero-Trust Server” (refer to Step 1 to 2 in Figure 4). Upon completing the transmission, the server needs to be notified again (refer to Step 7 to 8 in Figure 4). The zero-trust server maintains a record and monitors the frequency of grant transmissions within the communication pair to identify any anomalous behavior, such as brute-force (replay) attacks attempting to compromise the IoT ecosystem. The deployment of the control plane in an OTP^3^ IoT cybersecurity system, as illustrated in Figure 2, is optional.

#### 3.3.3. Data Plane

The data plane is designated for transfers during the operational stage of the system. Following the receipt of permission to initiate data transmission, the paired modules must independently generate a new and non-repeating OTP password, based on their SN (refer to Step 3 in Figure 4). Throughout the data transmission process, the paired modules utilize the OTP keys generated by the OTP password (refer to Step 4 in Figure 4) to perform encryption and decryption operations, respectively (refer to Step 5 to 7 in Figure 4). It is anticipated that there will be a proliferation of IoT devices in the future, and the designed data plane of OTP^3^ allows devices to directly exchange data through the zero-trust access virtual tunnel (refer to Figure 1). This approach enables the proposed OTP^3^ IoT cybersecurity system (refer to Figure 2) to distribute computing and storage resources to the endpoint devices, facilitating the switching of datagrams at the network edge to mitigate data transmission delays. This concept represents a novel realization of fog computing [1]. Consequently, the data plane is imperative for the proposed OTP^3^ mechanism and the OTP^3^ IoT cybersecurity system.

### 3.4. Practical Deployment Challenges of OTP^3^

The major deployment challenge of the OTP^3^ mechanism lies in Step 0 in Figure 4, where devices A and B are preset with an identical Serial Number (SN) for communication pairing. During the production or installation of IoT devices, engineers preset a series of non-repeating OTP Serial Numbers in the hardware of both paired devices A and B at both ends (e.g., in ROM). While this method requires additional engineering effort, establishing the pairing during device production is a crucial step toward achieving un-breakability based on Shannon’s theory [16]. Although the Diffie–Hellman [48] method used in Step 3 is presently the most commonly employed symmetric-key exchange technique, DH, however, is susceptible to cracking. With the ongoing advancements in computer processing power, such as quantum computing, even methods like eSTREAM [38] and Diffie–Hellman [48] may become vulnerable within a finite computation time. In essence, achieving cryptography security standards means that, with the current level of human technological prowess, future scientific developments are not limited in this aspect. Hence, to achieve the un-breakability based on Shannon’s theory [16], considering Step 3, using the OTP password exchanged on the public network, even if it is cracked and leaked, the OTP Serial Number preset in Step 0 on the device remains unknown to third parties (as it is not transmitted via the public network). Consequently, the OTP keys used in the encryption operation remain secure.

## 4. Design Implementation

**Figure 5:**
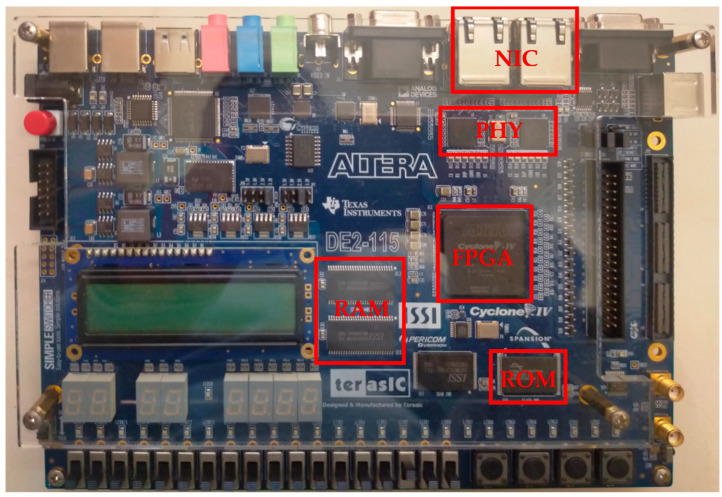
FPGA embedded-system development board [49].

This section commences with an introduction to the design and execution flow of the IoT device system architecture and data stream pipeline processing outlined in this paper. Subsequently, it details the integration approach of the micro-processor and hardware accelerators in the FPGA embedded-system board, as shown in Figure 5.

### 4.1. System Design Architecture

**Figure 6:**
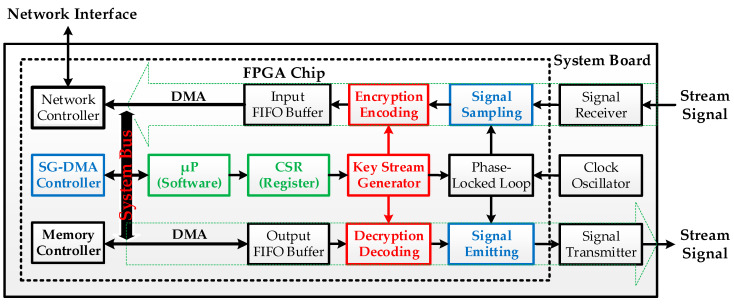
System architecture and pipeline data processing flow.

The system architectural design is depicted in Figure 6, which, above the light green arrow symbol, from the “Stream Signal” input to the “Stream Signal” output, encompasses multiple pipeline data processing units and associated control software programs, controlled by the internal micro-processor. Additionally, there is a “Network Interface” linked to a gateway for Internet data access. Several implemented hardware modules, including “Phase-Locked Loop (PLL)”, “Input/Output Buffers”, “NIC Controller”, and “Memory Controller” are utilized functional components provided in the FPGA design library [50]. Additionally, the self-designed hardware modules are indicated by blue and red boxed sections. The blue boxes represent functions including implemented high-speed “Signal Sampling” and “Signal Emitting”, and a “Scatter–Gather Direct Memory Access (SG-DMA) Controller” attached to the system bus, behind which the green box encompasses the “Micro-Processor (µP)” executing the related software control programs. Next are the “Configuration Status Registers (CSRs)” providing functional setting and execution status for the software control required by the designed hardware modules. Furthermore, the red boxes include modules of “Key Stream Generator”, stream data “Encryption Encoding”, and “Decryption Decoding”. In the subsequent sub-section, the system execution flow of data processing in the designed architecture of Figure 6 will be introduced.

### 4.2. System Execution Flow

**Figure 7:**
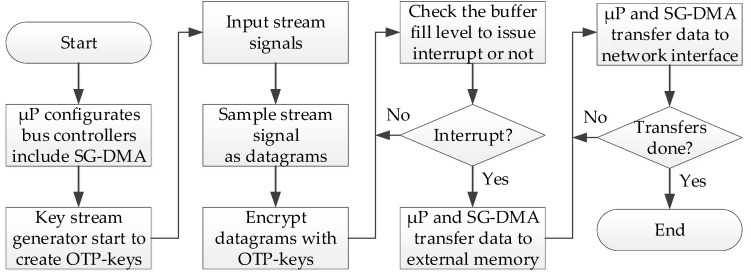
Data processing flow from stream signal to network interface.

In Figure 7, The processing flow of input stream signals is depicted by the green dashed arrow, as shown above in Figure 6. When the device begins to receive stream signals, the “Key Stream Generator” continuously generates OTP keys for real-time data encryption. The encrypted datagrams (i.e., ciphertext) are temporarily stored in the “Input FIFO Buffer.” This buffer can be configured to issue an interrupt signal to the micro-processor when it reaches a fill of half-full, near-full, or full capacity. The micro-processor then controls the SG-DMA controller to transfer the encrypted datagrams from the “Input FIFO Buffer” to the external memory on the system board. Conversely, datagrams from the Internet are first stored in the external memory, then the micro-processor can control the SG-DMA controller to move the encrypted datagrams (i.e., ciphertext) from the external memory to the “Output FIFO Buffer.” Subsequently, the datagrams are decrypted using the generated OTP keys and then emitted as stream signals.

Compared to merely utilizing software [51] or simulation [52] for computation and processing, the distinctive feature of this study lies in the integrated design of hardware and software. This effectively alleviates the computational burden on the micro-processor, enabling real-time streaming data encryption and decryption on IoT devices. Next, components of the FPGA system bus (depicted on the left side of Figure 6), encompassing the micro-processor, fundamental controllers, and application-specific key accelerators, will be expounded upon in the forthcoming sub-section.

### 4.3. FPGA Bus Components

**Figure 8:**
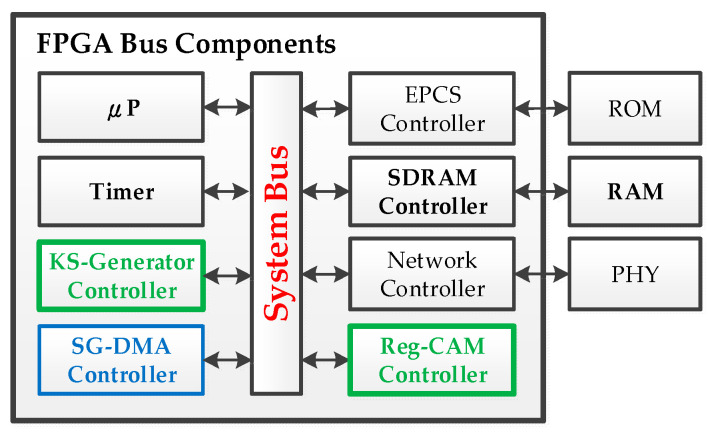
FPGA system bus components with external memory and network interface.

The system was implemented using the Intel Platform Designer [53] on an FPGA chip, as shown in Figure 8. An Intel Nios II micro-processor (µP) [54], synthesized on the FPGA, was utilized. The software was developed in the C language. Both the FPGA configuration file and micro-processor program were stored in the Serial Configuration Device (EPCS) Read-Only Memory (ROM), also known as bootloaders. A hardware “Timer” was integrated into the FPGA, enabling the micro-processor to retrieve precise timestamps for performance evaluations. The hardware timer operates simultaneously, without requiring additional processing in the micro-processor. Additionally, the system had TCP/IP protocol support and a network interface for remote control and data access.

To benchmark the performance against the “Key Stream Generator” in the pipeline data processing flow, the system designed the algorithm serving as the bus controller of Key Stream Generator (KS-Generator) controller. In particular, a Register-based Content-Addressable Memory (Reg-CAM) is used to accelerate the generation of the non-repeating key stream blocks. Lastly, a Scatter–Gather Direct Memory Access (SG-DMA) controller was designed using Verilog and synthesized as a system component connected to the system bus. These controllers allow the micro-processor to access relevant configuration and status registers, facilitating hardware and software cooperation. Specifically, the implemented controllers of Reg-CAM and SG-DMA are introduced as follows:

#### 4.3.1. Register-Based Content-Addressable Memory

Based on our understanding and the previous research [4], it is known that the traditional Non-Repeating PRNG (NR-PRNG) implemented solely in pure software cannot meet the efficiency requirements for real-time stream processing as proposed in this paper. Upon investigation, one feasible approach is to utilize Content-Addressable Memory (CAM) for data comparison by hardware acceleration [30]. Unlike traditional memory where the processor inputs an index address and the memory outputs the data byte stored at that location, CAM operates differently. In CAM, the processor inputs a data byte and determines whether this input data are already stored in the memory, outputting the result of the match operation. CAM implemented in FPGA can be categorized into two types: Register-based CAM (REG-CAM) and RAM-based CAM (MEM-CAM) [55]. We have integrated this REG-CAM module as part of the system bus (as indicated in the green box in Figure 8), providing the REG-CAM with a bus interface for the micro-processor to access its relevant control and status registers. This allows the micro-processor to input key stream blocks generated by the KS-Generator controller (as shown in the other green box in Figure 8) into the REG-CAM controller. Thees input data are then checked to determine if the key stream block has been used before. If it has, another key stream block is generated and tested until a non-repeating and usable key stream block is obtained. This key stream block is then stored to be used for encryption with the plaintext data stream, as elaborated in the subsequent Section 5 “Experimental Results”.

#### 4.3.2. Scatter–Gather Direct Memory Access

**Figure 9:**
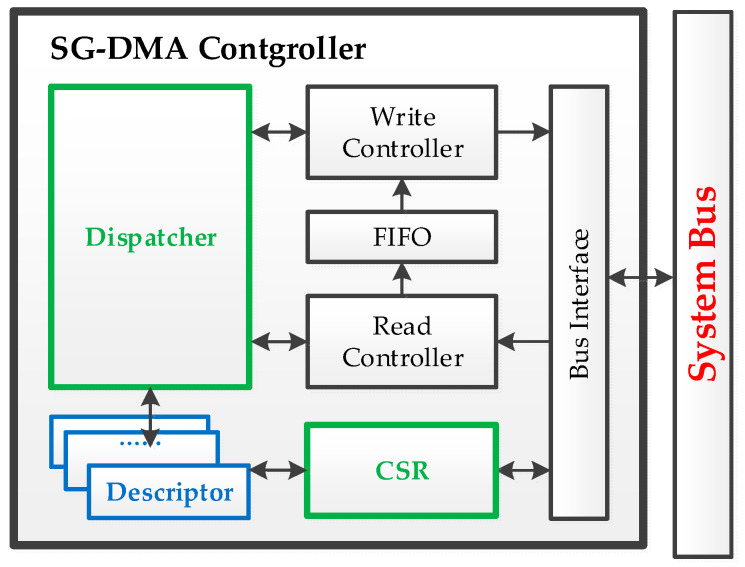
Design components of SG-DMA.

Moreover, the SG-DMA controller (as shown in the blue box in Figure 8) comprises an internal CSR, which the micro-processor can access to control SG-DMA operations as drawn in Figure 9. Upon receiving a start command from the micro-processor, the dispatcher fetches a descriptor, based on priority. These descriptors contain essential information for the read and write controllers to execute memory access for a specified length of a data block. An SG-DMA task involves the continuous execution of multiple descriptors. This functionality allows SG-DMA to effectively gather several small scattered data blocks into a larger data stream within a contiguous memory space, forming a substantial datagram, which can then be transmitted to the network’s physical layer (PHY).

**Figure 10:**
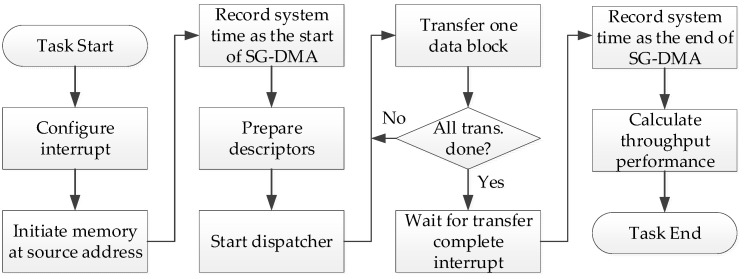
SG-DMA execution flow from the interrupt configuration to performance calculation.

The execution flow of an SG-DMA task is illustrated in Figure 10. For a fair comparison, the system time is recorded by a hardware timer (as a controller attached to the system bus shown in Figure 8 and described in Section 4.3. FPGA Bus Components). The performance calculation is from “Task Start” to “Task End” in Figure 10, including the total execution time, which consists of time required for programming the descriptors, starting the dispatcher, transferring data blocks, and executing the interruption of services. Moreover, test datagrams are first initialized as a sequential number (0, 1, 2, 3, …), as the assigned length of memory transfer by the micro-processor from a source address of the memory. After completion of the SG-DMA execution, the data at the destination address were verified to validate the correctness of execution. For more information regarding the performance metrics, the following sub-sections in Section 5 “Experimental Results” will provide the details.

## 5. Experimental Results

Building upon the system design architecture introduction, this section begins by conducting a performance analysis of the data stream pipeline with hardware processing. It then elaborates on the integration of the micro-processor and hardware accelerators within the FPGA. The experimental results first highlight individual software and hardware computations and subsequently engage in a discussion regarding the performance of software and hardware co-design.

### 5.1. Performance Analyses of Hardware Processing

**Figure 11:**
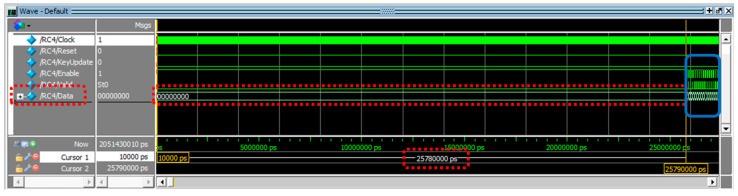
Initialization of S-Box memory and starting key stream generation for activating a hardware stream generator.

During the experiment, the “Key Stream Generator” first utilized the micro-processor to execute the RC4 algorithm, which was designed in pure software using the C language. Measurement of experimental data revealed that the initialization of the S-Box memory content took approximately 616 μs. Subsequently, generating ten thousand 8-bit key stream blocks required about 30,597 μs. As shown in the experimental results in Figure 11, in comparison, the “Key Stream Generator” designed in Verilog language took approximately 25.78 μs from reset to generating the first key stream block. This time is mainly used for initializing the content of the S-Box memory.dis

**Figure 12:**
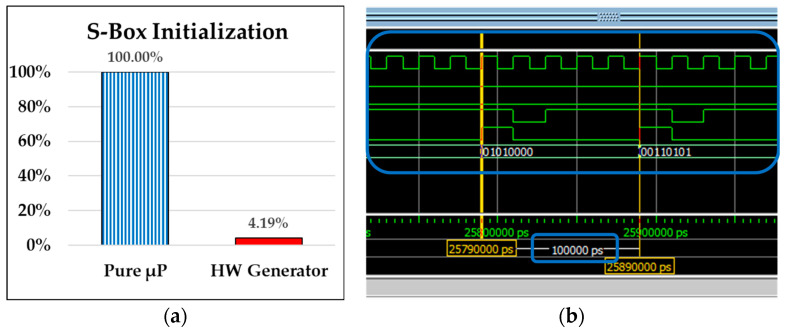
(**a**) Performance comparison depicted in percentage on the y-axis of pure software (Pure μP) processing and hardware key stream generator (HW Generator) on the x-axis for S-Box memory initialization. (**b**) Consecutive generation of multiple key stream blocks after activating the HW Generator.

The operating frequency of the FPGA development board used in this system is 50 MHz (i.e., 20 ns per clock cycle), indicating that the “Key Stream Generator” requires a startup time of 1289 clock cycles (=25.78 μs/(20 ns/cycle)). Figure 12a shows that, utilizing the “HW Generator”, the initialization of the S-Box memory content only requires 4.19% (25.78 μs/616 μs) of the time needed by “Pure μP” processing when that computation is performed by the micro-processor alone.

**Figure 13:**
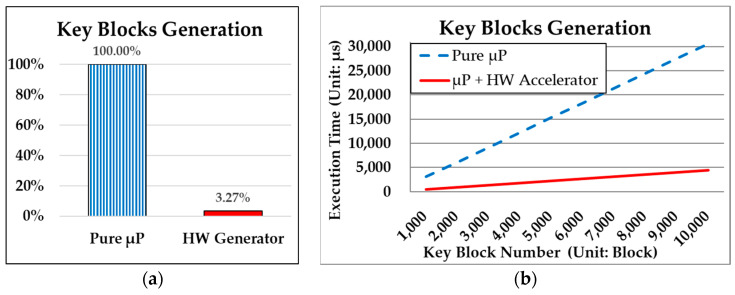
Performance comparisons of pure software (Pure μP) processing between (**a**) hardware generator (HW Generator) and (**b**) hardware and software co-design approach (μP + HW Accelerator) for generation of ten thousand key stream blocks.

After the system startup, as shown in Figure 12b in the solid blue box (a zoomed-in portion of key stream block generation from the previous Figure 11), a key stream block can be generated every 5 clock cycles of 100 ns (=20 ns × 5 = 100 ns = 100,000 ps), at the shortest. Accordantly, to generate ten thousand key stream blocks, it would take 50,000 clock cycles or an operating time of 1000 μs (100 ns × 10,000). Figure 13a evaluates the real system performance for generating ten thousand key blocks by “Pure μP” and “HW Generator”, respectively. It illustrates that the “HW Generator” consumes only 3.27% (1000/30,597) of the time required by “Pure μP” processing.

This sub-section illustrates the performance enhancement achieved through hardware processing of the “Key Stream Generator”, yet the key blocks generated by it could be repeated. The reuse of key blocks raises the risk of encrypted data being compromised, as discussed in the conventional cipher methods in Section 3.1 and Section 3.2. The significant contribution of this paper lies in achieving theoretical un-breakability, as proven by Shannon [16], through the proposed OTP^3^ secure mechanism, which requires non-repeating key blocks for data encryption. However, the non-repeating check is computationally intensive in the proposed OTP^3^ secure mechanism, as detailed in Section 3.3 and Section 3.4. Accordingly, the subsequent subsection will present experimental results to address this issue.

### 5.2. Performance Analyses of Hardware and Software Co-Design

**Figure 14:**
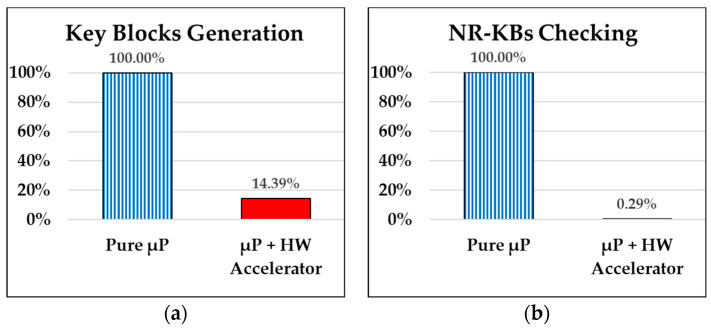
Performance comparisons of pure software (Pure μP) processing and hardware and software co-design approach (μP + HW Accelerator) for (**a**) generation and (**b**) non-repeating checking for ten thousand NR-KBs.

In comparison to the hardware generator of key stream blocks discussed in the previous section, we have also implemented the “KS-Generator” controller shown within the green box in Figure 8 on the FPGA system bus, using a hardware and software co-design approach (μP + HW Accelerator). This allows the micro-processor (i.e., µP in Figure 8) to read the results of the operation results from the “KS-Generator” controller. Figure 13b shows that generating ten thousand key stream blocks in the “μP + HW Accelerator” manner takes an average time of 4402 μs, which is only 14.39% (4402 μs/30,597 μs) of the average time required by “Pure μP” (30,597 μs) as indicated in Figure 14a. In other words, this approach can save over 85% of the micro-processor’s processing time.

Furthermore, to accelerate the processing speed of NR-PRNG for real-time stream processing, the performance of implemented REG-CAM controller introduced in Section 4.3.1 was evaluated. As shown in the experimental results in Figure 14b, when the micro-processor employs the REG-CAM controller (μP + HW Accelerator) to operate ten thousand key stream blocks for duplication checking, it takes 27,601 μs. This is only 0.29% (27,601 μs/9,371,161 μs) of the time (9,371,161 μs) required by pure software (Pure μP). The significant performance enhancement is attributed to the fact that as the number of generated key blocks accumulates, new key blocks must be compared to all previously used blocks. This leads to an increasing time to obtain a valid Non-Repeating Key Block (NR-KB). In this scenario, as the quantity of generated blocks accumulates, the use of REG-CAM for a hardware-accelerated comparison becomes even more effective compared to the time-consuming software-only approach.

**Figure 15:**
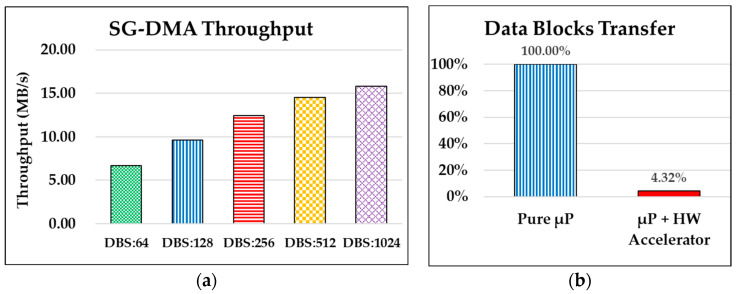
Performance comparisons of SG-DMA controller (**a**) among different data block sizes (DBSs) and (**b**) between pure software (Pure μP) processing and a hardware and software co-design approach (μP + HW Accelerator).

Next, to evaluate and demonstrate the performance of applying the implemented SG-DMA, we selected setting a different Data Block Size (DBS) for each operation of memory access. The DBS is a unit of burst memory access length used by the designed SG-DMA. That is, a memory read is continual for the assigned DBS, and then a memory write is also continual for the same DBS. Accordingly, the SG-DMA controller will support a First-In First-Out (FIFO) buffer to temporarily store the access data from a source memory address and then transfer them to another destination memory address. In experiments, we selected DBSs of 64, 128, 256, 512, and 1024 bytes. For a fair comparison, to transfer the same amount of data of 8192 bytes, the number of burst transfers for DBSs of 64, 128, 256, 512, and 1024 bytes are 128, 64, 32, 16, and 8 times, respectively. As Figure 15a shows, they are denoted as DBS:64, DBS:128, DBS:256, DB:512, and DBS:1024, respectively.

In Figure 15a, the throughput of DBS:64 is 6.66 MB/s, which is the lowest throughput performance in this experiment. Next, the throughput of DBS:128 is 9.64 MB/s, which is higher than that of DBS of 64 bytes (i.e., DBS:64). It is notable that the throughput of DBS:1024 is 15.83 MB/s, which is the best performance in this experiment. Overall, we observed that the memory accesses with a larger data block size came out with a better throughput performance. This is reasonable, because in addition to the memory access time between source and destination addresses and the bus turn-around time between transfers of data blocks, the SG-DMA execution overheads for each burst transfer for a DBS additionally include the programming efforts of µP, such as descriptor configurations, interrupt services executions, and so on. To transfer 8192-byte data in the memory, the number of burst transfers of DBS:1024 is 8 times, which is less than 128 times of DBS:64. Accordingly, the SG-DMA execution overhead of DBS:1024 (with 8 times of transfers) is less than that of DBS:64 (with 128 times of transfers). Consequently, the throughput performance of DBS:1024 overcame that of DBS:64 by around 238% (=15.83/6.66 × 100%).

Apart from the performance comparisons among different DBSs of SG-DMA, we furthermore transferred the 8192-byte data using pure µP programming (i.e., without SG-DMA) from the source to the destination addresses that are identical to those of the SG-DMA experiments in the previous sub-section. As Figure 15b shows, the throughput performance of the memory access by pure µP is a small value of 0.51 MB/s, which is less than the average throughput of 11.81 MB/s of SG-DMA. That means that only 4.32% ([0.51 MB/s]/[11.81 MB/s]) processing time of pure software (Pure μP) is required for µP, with the help of the SG-DMA controller. Accordingly, this experimental result demonstrates and validates the fact that a hardware accelerator (i.e., SG-DMA) for memory data movements is absolutely necessary for NB-IoT devices to generate the number of Transport Blocks (TBs) for accessing the 6G gNB.

In summary, this sub-section illustrates the performance advantages gained through the utilization of the hardware and software co-design approach (i.e., μP + HW Accelerator) in the proposed OTP^3^ secure mechanism. This enables the micro-processor (μP in Figure 8) to retrieve the key block generated by the “KS-Generator” controller, then performs a check on the generated key block using the REG-CAM controller (c.f. Section 4.3.1) to determine its repeating status. If the key block has not been used, it is stored for subsequent encryption with a plaintext data stream block. Finally, the micro-processor manages the SG-DMA controller (c.f. Section 4.3.2) to transfer the encrypted datagrams from the “Input FIFO Buffer” (c.f. Figure 6) to the external memory, preparing them for transmission to the Internet through the network controller. The system design architecture and execution flow are detailed in Section 4.1 and Section 4.2, respectively.

## 6. Discussion and Conclusions

In the future, with the increasing prevalence of NB-IoT and 6G networks, a multitude of IoT devices will be directly connected to the Internet using these technologies. These devices may not fall under the conventional internal network’s control, giving rise to a new set of information security challenges. Consequently, this study’s primary innovations and contributions can be summarized as follows:

- This paper introduces an FPGA-based implementation of a zero-trust secure stream data transmission system. It is designed for the transmission of inter-endpoint streaming signals, aligning with the fundamental principles of zero-trust networks.
- To prevent eavesdropping on transmitted information, this paper innovatively combines the principles of one-time passwords and one-time pads, creating the operational flow known as the One-Time Password Pad Process (OTP^3^) for secure transmissions between paired IoT devices.
- To tackle the substantial computational burden on the micro-processor, this paper introduces a mechanism in processing key block generations, non-repeating checks, and data block transfers for the stream pipeline and hardware accelerators.
- This hardware-accelerated and software co-designed approach effectively alleviates the workload on the micro-processor, thereby enhancing the overall system performance. Experimental results demonstrate remarkable throughput performance enhancements compared to traditional design schemes.

By harnessing the distinctive transmission characteristics and specific environments of IoT, such as the uninterrupted streaming signal exchange between devices and the preset two devices as a communication pair, this paper introduces OTP^3^. It is a secure mechanism tailored for transmitting streaming signals between IoT device endpoints within the zero-trust network framework. The implementation is carried out on an FPGA, incorporating hardware processing modules that facilitate real-time data stream pipeline processing. Empirical results affirm its efficiency in encryption processes and its capacity to handle substantial volumes of transmitted data within the data-plane of the Fog Computing architecture. Furthermore, the FPGA designs implemented in this system can be translated into an Application-Specific Integrated Circuit (ASIC), resulting in reduced production costs and lower power consumption for large-scale manufacturing. This represents a significant advantage for IoT devices, compared to methods employing Graphics Processing Units (GPUs), which are also utilized for accelerating data processing. Designing the proposed OTP^3^ operation flow with related functional components using both ASIC and GPU approaches necessitates substantial engineering effort. However, it is worthwhile undertaking this task, as conducting a more comprehensive comparison will be instrumental in validating the performance of the proposed solution, contributing to the next phase of advanced progress in this research.

Another future endeavor involves a collaborative design with a Quantum Key Distribution (QKD) key exchange method [56], like the pioneering BB84 QKD protocol [57]. This approach allows two parties to establish a shared encryption key through a quantum channel, known as a QKD link [58]. In the event of an eavesdropper’s attempt to intercept the key, quantum principles will enable the communicators to detect such intrusion. QKD offers a solution to the security challenges posed by the Diffie–Hellman (DH) algorithm [48], used for public key exchange on the Internet, particularly in anticipation of future threats, including those associated with Quantum Computing. Therefore, further empirical validations in real-world scenarios, particularly under diverse and challenging conditions, are worth pursuing as future works with respect to this paper.

## Data Availability

Data is contained within the article.

## Abbreviations

µP	Micro-processor
AES	Advanced Encryption Standard
ASIC	Application-Specific Integrated Circuit
BB84	A Quantum Key Distribution Scheme by Bennett and Brassard in 1984
CAM	Content-Addressable Memory
CoAP	Constrained Application Protocol
CSR	Configuration Status Registers
DBS	Data Block Size
DES	Data Encryption Standard
DH	Diffie–Hellman
D-OTP	Double OTP
DTLS	Datagram TLS
EPCS	Serial Configuration Device
FIFO	First-In First-Out
FPGA	Field-Programmable Gate Array
gNB	Basestation of 5G
GPU	Graphics Processing Unit
HW	Hardware
ICS	Industrial Control System
IETF	Internet Engineering Task Force
IIS	Industrial Information System
IoT	Internet of Things
IP	Internet Protocol
JPL	Jet Propulsion Laboratory
KS-Generator	Key Stream Generator
M2M	Machine-to-Machine
MEC	Multiaccess Edge Computing
MEM-CAM	RAM-based Content-Addressable Memory
mMTC	Massive Machine-Type Communications
NASA	National Aeronautics and Space Administration
NB-IoT	Narrow Band Internet of Things
NIC	Network Interface Controller
NIST	National Institute of Standards and Technology
NR-KB	Non-Repeating Key Block
NR-PRNG	Non-Repeating PRNG
OTP	One-Time Password
OTP^3^	One-Time Password Pad Process
PHY	Physical Layer
PLL	Phase-Locked Loop
PRNG	Pseudo Random Number Generator
PSA	Platform Security Architecture
QKD	Quantum Key Distribution
RC4	Rivest Cipher 4
Reg-CAM	Register-based Content-Addressable Memory
ROM	Read Only Memory
SG-DMA	Scatter–Gather Direct Memory Access
SDN	Software-Defined Networking
SN	Serial Number
SSL	Secure Sockets Layer
TB	Transport Block
TCP	Transmission Control Protocol
TLS	Transport Layer Security
TMIS	Trend Micro IoT Security
UE	User Equipment
VNF	Virtual Network Function
WEP	Wired Equivalent Privacy
WPA	Wi-Fi Protected Access
